# The Impact of Industrial Odors on the Subjective Well-Being of Communities in Colorado

**DOI:** 10.3390/ijerph15061091

**Published:** 2018-05-28

**Authors:** Mohamed A. Eltarkawe, Shelly L. Miller

**Affiliations:** Mechanical Engineering Department, University of Colorado Boulder, Sustainability, Energy and Environment Complex, East Campus, 4001 Discovery Drive, Boulder, CO 80303, USA; Mohamed.eltarkawe@colorado.edu

**Keywords:** evaluative well-being, air quality, Globeville, Elyria Swansea, hedonic well-being

## Abstract

Odor pollution was identified as a top priority of the community of North Denver. Previous studies that investigated the impact of air pollution in North Denver focused on adverse health effects, rather than mental well-being. This study assessed the impact of odors from industrial sources on the subjective well-being (SWB) of North Denver residents, and of four similar communities in Colorado for comparison. An online survey was sent to participants from Greeley, Fort Collins, Fort Lupton, North Denver, and Pueblo, asking questions about SWB and odors in their areas (*n* = 351). The evaluation of SWB was performed using a novel approach that appraises three aspects of SWB. This approach of evaluating SWB has not been used in odor exposure studies. A proportional odds logistic regression model was used to estimate nine measures of SWB. The results showed that participants who reported that the air is very fresh or the odor is highly acceptable had higher levels of SWB. This association suggests that residents who live in areas exposed to strong industrial odors had lower levels of SWB. A subset of participants in this study took the survey four times in one year. Longitudinal analysis showed that evaluative satisfaction was slightly associated with seasonality. Both satisfaction with how life turned out and satisfaction with standards of living slightly increased during the fourth quarter of the year. The study also found that four of the nine measures can be used to represent SWB in future studies. Two of those measures were evaluative SWB, and the other two were positive hedonic SWB measures. A comparison between the five communities showed that well-being levels in North Denver and Greeley were not significantly different than those in Fort Collins or Fort Lupton. The comparison, however, showed that Pueblo had the lowest levels of well-being among all communities.

## 1. Introduction

The residents of the northern part of the Denver metropolitan area frequently experience odors from commercial and industrial businesses, two major highways, railyards, and utilities. Globeville and Elyria Swansea are probably the most affected neighborhoods in North Denver, in which over 70% of their areas contain these types of businesses. The residents of both neighborhoods, who are predominantly Hispanic (Globeville is 68% and Elyria Swansea 84% Hispanic) have a long history of odor complaints. Since 2004, Denver City has received 1322 odor complaints [1]. According to the 2014 Globeville and Elyria Swansea Health Impact Assessment Report [2], odor pollution was identified as a top priority of the community of Globeville and Elyria Swansea. One example of the odors experienced in Globeville is an intermittent and unpredictable coal tar odor that can cause burning eyes and throat, headaches, skin irritation, and sleep problems. In response to odor complaints and concerns in North Denver, a University of Colorado Boulder study, funded by an environmental justice grant from the U.S. Environmental Protection Agency (EPA), was conducted with the Globeville community in 2012. The aim was to address the odor issue by attempting to identify pollutants responsible for the coal tar odor and to link the odor with pollutants emitted from specific facilities [3]. The EPA study concluded that coal tar odors were frequently reported and high levels of naphthalene were measured when the winds came from the Northwest, bringing emissions from the creosote facility, Koppers Inc., into the community. However, all pollutant concentrations were below the odor and toxicity thresholds. The study recommended a more detailed investigation to explain the effects of all industrial odors in Globeville and the surrounding communities, and to assess the relationship between odor exposure and well-being.

Understanding the psychological impact (impact on quality of life) of odor exposure, in particular odors from industrial sources, in the nearby residential communities is challenging [4,5]. Assessment of this relationship is difficult because both the exposure and response are hard to measure and there is no universal technique that could be applied to evaluate them. Odors are a complex mixture of chemicals and most of them have potential odorous impacts at very low concentrations [6]. The strength of odors in ambient air is associated with the concentrations of the chemical components, mainly volatile organic compounds (VOCs) [7]. Studies show that odors cause nuisance and acute health effects such as headaches, nausea, and eye and throat irritation [8]. Exposure to intensive odors increased inflammation in the nose and respiratory area [9], and caused a histamine release [10]. In some cases, odors can even cause death due to toxic compounds [11].

Odors are characterized with instrumental or odor sensory methods. The instrumental methods measure the chemical components of odors in the air, typically using gas chromatography/mass spectrometry [12] or photoionization detectors [13]. The sensory method (sometimes called perceived air quality) involves a human panel to assess air quality and/or odor exposure. Odor sensory methods are often more helpful than instrumental methods in many applications, because they do not have a limit of detection issue and reflect the actual human perception of odor. Bereznicki et al. [14] compared perceived odors and chemical emissions from dairy and swine facilities and found the two methods were comparable. Many studies preferred perceived air quality assessment in indoor environments [15,16,17,18,19,20,21]. It has also been used in outdoor applications [6,22,23].

Using standardized questionnaires for odor exposure assessment is common and reliable. A six-point scale question about odor intensity (odor strength from 1 = very slight to 6 = extremely strong) and a nine-point scale question about hedonic tone (pleasant and unpleasant odor), in addition to odor frequency data, were used in an odor exposure assessment study [24]. In a study about the association between environmental odor exposure from a fertilizer plant for mushroom cultivation and somatic symptoms [25], a standardized survey was used to evaluate the degree of odor annoyance (eleven-point scale). Luginaah [26] used community health surveys to evaluate the changes in odor perception and odor annoyance between 1992 and 1997. Anonymous questionnaires were used to evaluate the degree of perceived annoyance in a study that investigated the relationship between outdoor air pollution from livestock facilities and perceived odor annoyance [4]. The degree of annoyance was assessed by a five-point scale question, ranging from 0 (not annoying) to 4 (extremely annoying). Pedersen [27] used a five-point scale from 1 (do not notice) to 5 (very annoyed) to assess odor exposure.

Both academic and policy circles have showed an increased interest in the impact of quality of life recently, which is a catch-all term used to describe the health, comfort, and happiness experienced by an individual. Many aspects need to be addressed when measuring quality of life (e.g., economic, subjective, objective, and environmental components). Subjective well-being (SWB) is one of the most frequent methods used to measure quality of life, which is often done using surveys. Well-being is defined most simply as the state of being comfortable, healthy, or happy.

Satisfaction and happiness are widely used to measure subjective well-being [27,28,29,30,31,32,33]. However, recent studies required a distinction between two important aspects when subjective well-being is evaluated [34,35,36,37,38]. The first aspect is evaluative well-being, EWB (sometimes called global well-being or life evaluation), which can be measured by the overall judgment of life, such as general life satisfaction and general happiness. The second aspect is hedonic well-being, HWB (feelings and mood), which can be measured by experienced happiness, enjoyment, stress, or sadness. In addition, hedonic well-being is often divided into positive hedonic well-being (+HWB) and negative hedonic well-being (−HWB) [36,37]. According to Hicks et al. [37], when subjective well-being data are collected, these three aspects of well-being (EWB, +HWB, and −HWB) should be reflected.

All studies that have been conducted in the North Denver area mainly focused on linking air pollution or industrial odor pollution to odor sources or adverse health effects, not well-being [3,39]. There are, however, a few studies that investigated the impact of industrial odors on well-being in other locations. The focus of some of these studies was mainly on the impact of odor exposure on psychological stress. A study on the psychological impact of malodors from a mushroom fertilizer production plant found that levels of cortisol—a steroid hormone released in the human body in response to stress—are correlated with odorant exposure [40]. Luginaah et al. [26], in a study on the effects of odors from a petroleum refinery in Oakville, Ontario, found that the decrease in odor exposure led to a decrease in negative perception and concerns. Moreover, the same study found an association between psychological reaction to environmental stress and odor exposure. The association between stress and odor exposure was also confirmed in a cross-sectional study in animal research [41]. There is compelling evidence in the literature supporting that long-term psychological stress leads to diseases such as depression, and cardiovascular disease [42]. Recent research found a link between stress and human immunodeficiency virus (HIV) progression to acquired immunodeficiency syndrome (AIDS) [43].

Other studies focused on the impact of odor exposure (mainly from animal production) on mental state indices such as depression, anger, confusion, tension, fatigue, and ability to focus. Schiffman et al. [44] reported an association between unpleasant odors (from large hog operations) and mood state. The residents who were exposed to the strong swine odors were most likely to have higher levels of depression, anger, fatigue, confusion, and tension when compared with residents who were not exposed to such odors. This is supported by the findings of Nordin et al. [45], who found that unpleasant odors had a negative impact on ability to focus in a study on 55 young adults. In a field study, odor exposure from a pig production facility was correlated with increased annoyance and symptoms. The study also concluded that odor exposure could be a risk factor for lower well-being and adverse health [25].

**Figure 1:**
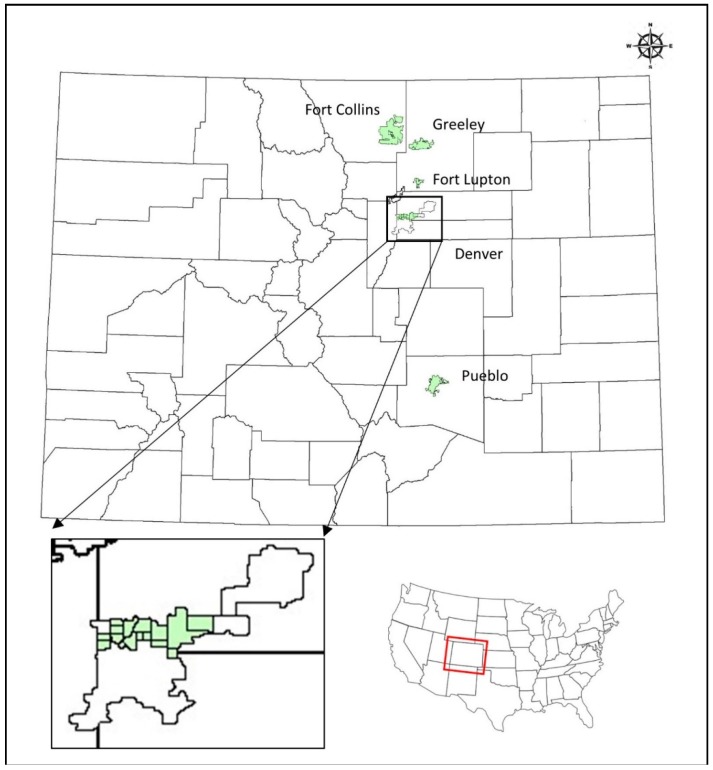
Locations of the five communities: Fort Collins in Larimer County, Greely and Fort Lupton in Weld County, North Denver in Denver County, and Pueblo in Pueblo County.

This study investigates whether odors from industrial sources impair the subjective well-being (SWB) of residents in the often-low-income surrounding communities in Colorado. The study expands the scope and includes all north of Denver metropolitan neighborhoods (Sloan Lake, Wheat Ridge, West Highlands, Highlands, Chaffee Park, Sunny Side, Globeville, Elyria Swansea, Cole, Downtown, Northeast/North Park Hill, Montbello, Stapleton, E. Colfax, Aurora, and Commerce City). In addition, four other Colorado communities were chosen outside of Denver for comparison purposes. Three of them are demographically similar to Globeville and Elyria Swansea (disproportionately Hispanic, low household income, and low education). These three communities are Greeley, Fort Lupton, and Pueblo. Unlike these three communities, Fort Collins was selected to be different. Figure 1 shows the locations of the five communities, and Table 1 details some of the demographic information of the five communities [46,47,48]. This study closely investigates the impact of odor exposure on SWB using surveys. The evaluation of SWB is performed using the recent approach of the three aspects of subjective well-being (EWB, +HWB, and −HWB). Odor exposure, on the other hand, is evaluated using two odor-related variables; namely perceived odor and odor acceptability. To the best of our knowledge, this recent approach of evaluating SWB has not been used in odor exposure studies.

## 2. Materials and Methods

### 2.1. Study Design

A 60-question online survey was designed to collect data from the five Colorado communities. The online survey covers aspects related to SWB (48 questions), odors (5 questions), and demographic information (7 questions). The SWB part of the survey is a widely-used and validated survey that was adopted from the New Economics Foundation (NEF) in the United Kingdom (see the complete survey in Supplementary: Survey S1) [49].

The recruitment of study participants was done through contacts, flyer distribution, government entities, non-profit organizations, student clubs, and social media. Participation in the study was completely voluntary and participants could withdraw from it at any time without penalty or consequences. We recruited adult participants aged 18 or older, and all were required to consent before participating in the study. Any personal information was removed and the data were coded and kept confidential. Upon consent, the participants were given access to the online survey.

The last question in the online survey asked the participant to provide an email address to be contacted for further steps. The participants were asked to take the survey four times on a voluntary basis during the one-year study period (once every three months).

This study was approved by the University of Colorado Boulder’s Institutional Review Board, Protocol #16-0065.

### 2.2. Variables of Interest

The purpose of the online survey data collection was to quantify any relationships between SWB and the independent variables (demographics and odor-related characteristics). To measure SWB, three aspects of SWB were used: evaluative well-being (EWB), positive hedonic well-being (+HWB), and negative hedonic well-being (−HWB) [35,36,38]. Each well-being aspect was measured by three questions from the online survey. EWB is a measure of general feeling, which includes the following: the participant is overall happy, the participant is satisfied with how life turned out, and the participant is satisfied with their standard of living. +HWB is a measure of recent positive feeling, which includes the following: the participant is satisfied with life nowadays, the participant enjoyed life last week, and the participant was happy last week. −HWB is a measure of recent negative feeling, which includes the following: the participant felt depressed last week, the participant could not get going last week (powerlessness), and the participant felt sad last week. The nine measures mentioned above were selected for this project based on the literature. They were selected to cover the three aspects of SWB used in many recent studies, with a focus on happiness and satisfaction [34,35,36,37,38]. All dependent variables were arranged from lower well-being to higher well-being scores. For example, recent happiness was arranged from *none of the time I was recently happy* to *all the time I was recently happy*. Similarly, recent depression was arranged from *all the time I was depressed* to *none of the time I was depressed*. In the results section, the selection of these nine measures is compared with a selection based on principal component analysis (PCA).

As explanatory (independent) variables, two odor-related variables of interest were chosen, as well as three demographic variables. The first odor-related variable is perceived odor, with response categories of *air is very fresh, air is fresh, air is neutral, air has strong odor,* and *air has very strong odor*. The second odor-related variable is odor acceptability, with response categories of *low acceptability*, *middle acceptability*, and *high acceptability*. Three binary demographic variables were considered as independent variables: employment (*Yes, No*), age (*45-year-old or below, Above 45*), and gender (*Male, Female*). 

### 2.3. Statistical Analysis

#### 2.3.1. Principle Components Analysis

Principal Component Analysis (PCA) is a widely-used data reduction procedure by which the matrix of possibly correlated variables is decomposed into eigenvectors. These eigenvectors will then be sorted based on the corresponding eigenvalues. Following the authors of [50], only eigenvectors with corresponding eigenvalues greater than or equal to one were considered. In the field of psychology, it is expected to have correlation between components. Therefore, it is more realistic to use the oblique rotation (oblimin) instead of orthogonal rotation (varimax). Data suitability procedures for factor analysis were carried out based on sample size and sample-to-variable ratios [51,52,53], Kaiser–Meyer–Olkin Test [54], Bartlett’s Test [55], and correlation matrix (factorability) [56].

#### 2.3.2. Chi-Squared and Ordinal Logistic Regression

The chi-squared test was used to explore relationships between the dependent variables (EWB, +HWB, and −HWB measures) and the independent variables (employment, gender, age, perceived odor, and odor acceptability). An ordinal logistic regression model was also applied to quantify the associations between the variables.

An ordinal logistic regression is a model that can be used when the dependent variable is ordinal in nature, for example, the amount of time a person feels happy can be classified as *all the time*, *most of the time*, *some of the time*, and *none of the time*. The proportional odds regression was used in this study and it is one of the most popular and widely-used models among the ordinal regression models [57,58,59]. Confidence intervals of 95% were used to indicate whether significant association exists. The Lipsitz test was used to assess the goodness-of-fit for the ordinal logistic regression [57].

#### 2.3.3. Composite Scoring 

The Center of Well-being at NEF recommends using three sets of well-being questions. The first set is the Office for National Statistics (ONS) subjective well-being questions. This set of questions was added to the Integrated Household Survey (IHS) in April 2011. The IHS is a composite survey consisting of four questions that collects data from more than 200,000 people in United Kingdom (for ONS questions, see Table S1). The Short Warwick–Edinburgh Mental Well-being Scale (SWEMWBS) is the second set. It contains seven questions and each has five outcomes (see Table S2). These questions, designed by Warwick and Edinburgh University in 2006, are a validated and reliable measure of flourishing positive mental well-being (Health Survey for England, 2011). The third set contains a single question about social trust that is not a direct measure of SWB, but it is a major driver of overall SWB—see Table S3 [49]. One advantage of NEF’s well-being survey, which we used in our study, is that the survey contains all of the ONS questions, five questions of SWEMWBS, and the social trust question. Two questions from the SWEMWBS set are not available in NEF’s survey (ability to think clearly and to make up your mind about things). Both questions were ignored when we calculated the composite score of SWEMWBS. Social trust measure was excluded as it is not a direct measure of SWB.

Another advantage of NEF’s SWB survey is that the survey questions can be divided into SWB explanatory groups and SWB measures. The SWB explanatory groups include: social (four questions), employment (seven questions), health (three questions), appreciation (five questions), and freewill (two questions). In addition to these groups from NEF, our survey includes an odor-related group (four questions) and demographics (seven questions), which were added to the survey as a part of the study design.

## 3. Results and Discussion

### 3.1. Sample Size

Four different approaches were compared to determine the sample size in our study. First, a power analysis for an ordinal logistic regression was conducted to determine a sufficient sample size using an alpha of 0.05, power of 0.95, odds ratio of 1.3, and one-tail test. The analysis assumes that all dependent variables are log-normally distributed. Based on those assumptions, the desired sample size is 221. Secondly, we calculated the sample size using Whitehead’s formula, which is based on the proportional odds model. Using this formula, the maximum sample size among the nine dependent variables is 192 participants [60,61,62]. A third method involves using Hmisc R Package (R Core Team (2017). R: A language and environment for statistical computing. R Foundation for Statistical Computing, Vienna, Austria) with two power values (0.90 and 0.80) resulting in a maximum sample size of 280 (Power = 90%) and 209 (Power = 80%). The last method we used to determine sample size involves using a rule of thumb. The number of event per variable is at least 20–50, as suggested by van der Ploeg et al. [63]. Therefore, our five-level independent variable model indicates that 100–250 participants are required as a sample size. Based on the four approaches above, we can conclude that our experiment sample size (326) is well-above all calculated sample sizes, and thus it is sufficient for our model analysis. 

### 3.2. Using Principal Component Analysis to Reduce Well-Being Data and Compare with Well-Being Measures Selected Based on the Literature

The survey contains 24 (out of 60) questions that can directly measure well-being [49]. Principal Component Analysis (PCA) was used to reduce these 24 questions of well-being while retaining as much as possible the variance in the data. Four significant components (those whose eigenvalue is higher than 1) were selected, accounting for 59.78% of the variance. The oblique rotation of standardized component loadings shows that the data load clearly on four components. As shown in Table 2, component 1 describes the loading of positive well-being items (both evaluative and hedonic) and it accounted for 42.4%. Component 2 accounted for 6.6% and is characterized by negative well-being items. Components 3 and 4 accounted for 5.8% and 4.97% of the variance, respectively. They are characterized by other well-being items that are related to being tired and trust. The results from the PCA analysis are in good agreement with the nine selected measures of well-being. Six of the nine measures fall in the group of items loaded on component 1. All six measures are positive well-being measures. The other three selected well-being measures fall into group two, where items loaded on component 2. The three measures are recent negative well-being measures.

PCA was also performed on the nine selected well-being measures themselves. Table 3 shows that three components explain 77% of the variance. The oblique rotated solution effectively separates the data into evaluative positive well-being items loading on component 1, negative hedonic well-being items loading on component 2, and positive hedonic well-being items loading on component 3. The question, *all things considered, how satisfied are you with life as a whole nowadays?,* loaded on component 1 with the evaluative well-being items. This is not totally unexpected as nowadays could be thought of as an extend period of time other than recent time. The classification of the nine measures into three groups agrees with the classification from the literature.

### 3.3. Chi-Squared Test between SWB Measures and the Independent Variables

Table 4 shows a summary of the Chi-squared test for independence between all SWB measures and the independent variables. Seven SWB measures were associated with employment, while six measures were associated with gender. The binary age variable did not show significant association with SWB measures, except with recent powerlessness. Both perceived odor and odor acceptability associated with all EWB measures, recent satisfaction, and recent sadness.

**Figure 2:**
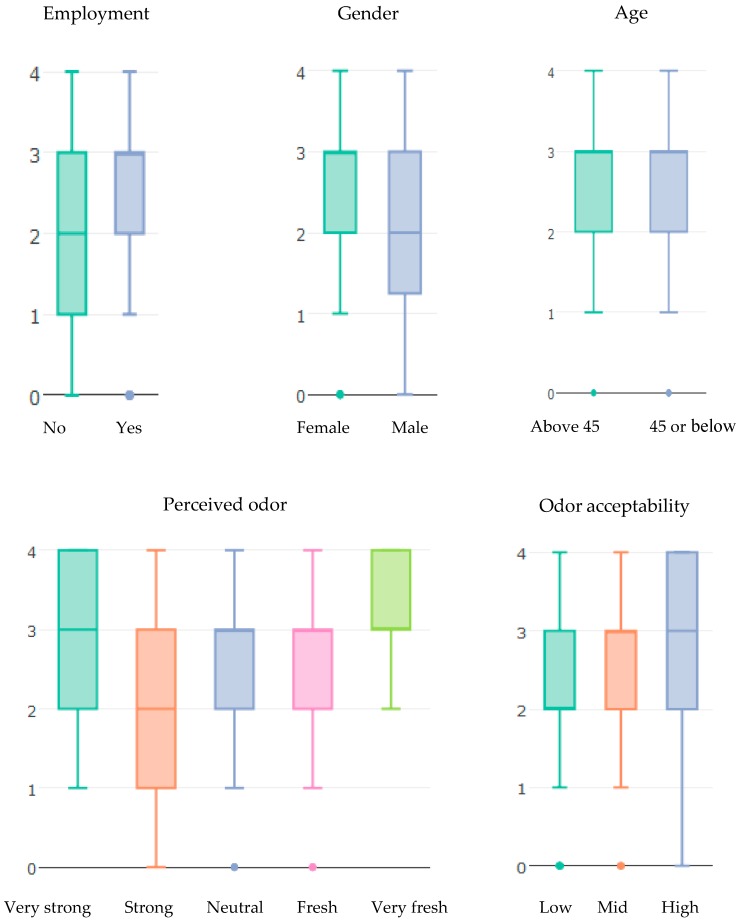
The effect of employment, gender, age, perceived odor, and odor acceptability on one subjective well-being (SWB) measure: satisfaction with how life turned out. *Y*-axis is the score of satisfaction with how life turned out, where 4 indicates the highest level of satisfaction and 0 indicates the lowest level of satisfaction.

Figure 2 shows boxplots of one SWB measure (satisfaction with how life turned out) for each independent variable. The difference between employed and unemployed participants is statistically significant ($\chi^{2}\left(4\right)=16.52,\;p<0.002)$ in favor of employment. Female participants had statistically significant associations with higher satisfaction with how life turned out than male participants $(\chi^{2}\left(4\right)=12.1,\;p<0.02).$ No significant difference was found between the *Above 45 years old* group and the *45 or below* group $\left(\chi^{2}\left(4\right)=3.95,\;p<0.41\right)$. We found statistically significant differences between perceived odor outcomes $\left(\chi^{2}\left(16\right)=30.35,\;p<0.016\right)$ and between odor acceptability outcomes $(\chi^{2}\left(8\right)=16.85,\;p<0.03).$ The chi-squared test does not quantify the association between satisfaction with how life turned out and the independent variables (employment, gender, perceived odor, and odor acceptability). To quantify the association between the nine SWB measures and the independent variables, an ordinal logistic regression was used. 

### 3.4. Ordinal Logistic Regression Results

A total of 351 participants joined the study and submitted the online survey (100 from Greeley, 40 from Fort Lupton, 62 from North Denver, 117 from Pueblo, 22 from Fort Collins, and 10 from LaSalle). This number of participants was reduced to 326 by excluding surveys from outside the five communities and repeated surveys when they were identified. The weighted sample was 25% male and all participants were at least 18 years of age (19% aged 18–25, 37% aged 26–35, 26.5% aged 36–45, 11% aged 46–55, 5.5% aged 56–65, 0.5% aged 66–75, and 0.5% aged 76–85). To avoid the effect of small numbers of participants in certain groups of age, the variable age was dichotomized at the median [64].

**Figure 3:**
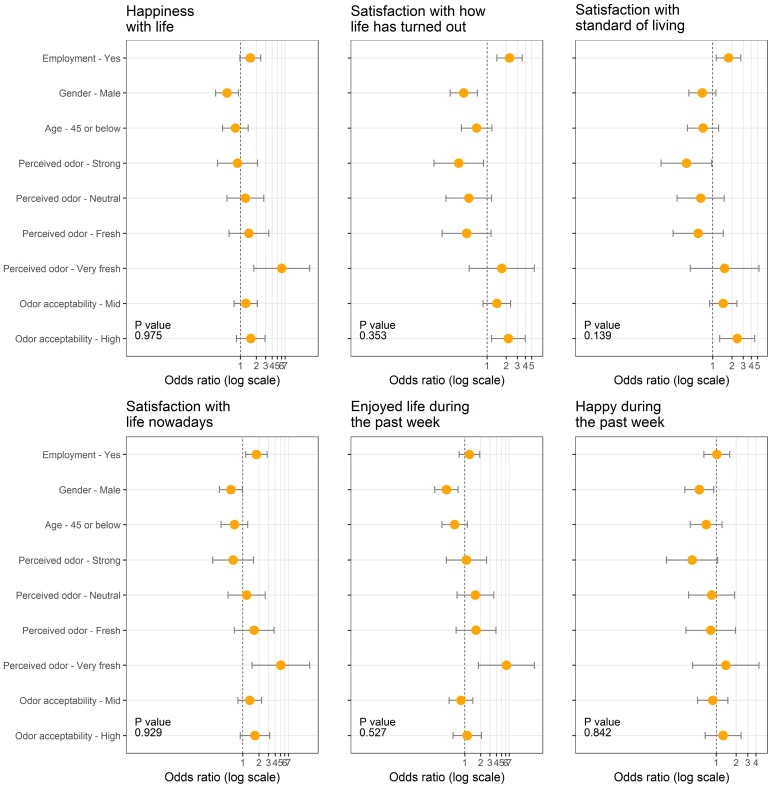

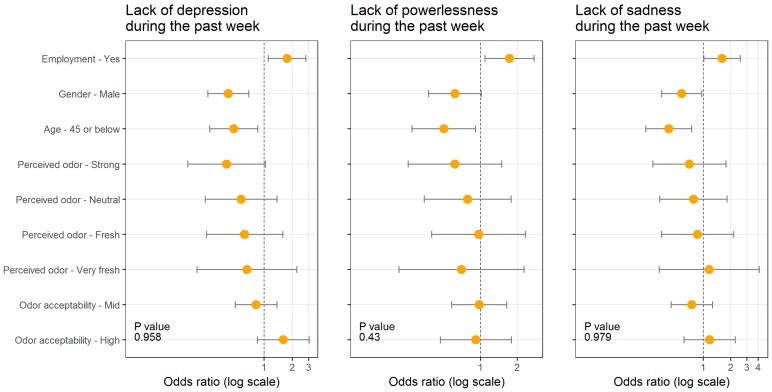
Forest plots of the effects of the five independent variables on the measures of subjective well-being.

The proportional odds logistic regression model was used to estimate associations between the five independent variables and measures of SWB at the conventional 5% level of significance. The results from the proportional odds model are obtained in the form of odds ratios (ORs), which are presented graphically as *forest plots* in Figure 3. The values on the *x*-axis are the ORs obtained from the proportional odds regression model output. The increase in OR values in the forest plots indicates better SWB and confidence intervals excluding one are considered significant.

#### 3.4.1. Well-Being and Employment

The proportional odds model results indicate that the three measures of satisfaction and −HWB measures were associated with employment. The OR of 2.25 (95% CI = 1.43–3.56), OR of 1.76 (95% CI = 1.14–2.74), and OR of 1.79 (95% CI = 1.13–2.84) suggest that employed participants tended to have higher levels of satisfaction with how life turned out, satisfaction with standards of living, and recent satisfaction, respectively. Similarly, the OR of 1.76 (95% CI = 1.11–2.79), OR of 1.73 (95% CI = 1.09–2.76), and OR of 1.60 (95% CI = 1.03–2.54) suggest that employed participants reported higher levels of lack of recent depression, recent powerlessness, and recent sadness, respectively. It is probably worth mentioning that *higher levels of lack of a well-being (WB) measure* has the same meaning as *lower levels of that WB measure*. These results are in good agreement with the strong evidence in the literature of the negative impact of unemployment on SWB [28,30,31,32,65,66,67]. Other studies that measured SWB based on psychological distress and depression [68,69] also showed that SWB was negatively correlated with unemployment. Those results are also in good agreement with our findings that indicate lower levels of −HWB are correlated with employment.

#### 3.4.2. Well-Being and Gender

Male participants tended to have lower levels of SWB. Precisely, male participants had lower levels of general happiness (OR of 0.56; 95% CI = 0.34–0.92), and satisfaction with how life turned out (OR of 0.43; 95% CI = 0.27–0.70). Measures of +HWB are significantly associated with gender. The OR of 0.60 (95% CI = 0.37–0.98), OR of 0.45 (95% CI = 0.27–0.75), and OR of 0.56 (95% CI = 0.34–0.92) indicate that males had a lower level of recent satisfaction, recent enjoyment, and recent happiness, respectively. Male participants also had higher levels of recent depression (OR of 0.41; 95% CI = 0.25–0.68) and recent sadness (OR of 0.58; 95% CI = 0.35–0.97).

According to the literature, the gender impact on SWB seems to lack consistency. A number of studies [68,70] showed that gender has no effect on SWB, while other studies showed that gender correlated with SWB. Some studies that found females are more likely to have higher well-being than males [71,72]. In contrast, other studies found that women have lower levels of SWB [73], and higher levels of stress, worry, and sadness [36], and tend to be more critical of themselves than men [65]. The same study by Stone et al. [36] found that women reported higher evaluative well-being. A study conducted by Wu et al. [74] investigated mental health and suicidal thoughts among 1848 pilots, and concluded that higher levels of depression were found among pilot women than among pilot men. The same study also found that women tended to report more days of poor mental health than men. According to the U.S. Centers for Disease Control and Prevention, women experience depression more than men in the United States. Flatau et al. [32] found that both incidences and attempts of suicidal thoughts were higher among women.

#### 3.4.3. Well-Being and Age

The binary variable of age, *45 years old or below* and *Above 45 years old*, showed significant association with only the measures of −HWB. The participants who were aged 45 or below had nearly half the odds of those who were above 45 years old. The OR of 0.47 (95% CI = 0.26–0.85), OR of 0.50 (95% CI = 0.27–0.91), and OR of 0.42 (95% CI = 0.24–0.75) indicate that participants aged 45 years old or below had higher levels of recent depression, recent powerlessness, and recent sadness, respectively. The measures of both EWB and +HWB did not show significant association with binary age. We decided to use a dichotomized age variable because the number of participants in some age groups was not sufficient [64]. The weighted sample of the binary variable was 82% aged 45 years old or below and 18% above 45 years old. Our findings about −HWB with the binary age seem to be consistent with the findings of Steptoe et al. [35], who found that elderly people, despite their health challenges, seem to experience less stress, worry, and anger. Typically, however, in the studies that investigated the impact of age on SWB, age is used as a continuous variable. Most of those studies indicated that age has a U-shaped effect on EWB, particularly on general happiness [28,30,35,65,75,76]. Our findings are still in partial agreement with the U-shaped results, as the EWB and +HWB increase after the age of 50 years old [35,65].

#### 3.4.4. Well-Being and Perceived Odor 

Almost all measures of EWB and +HWB showed a strong association with perceived odor, while −HWB measures did not show significant association. The OR of 6.06 (95% CI = 1.80–20.40), OR of 5.08 (95% CI = 1.49–17.33), and OR of 6.16 (95% CI = 1.81–20.91) suggest that the participants who reported that the air was very fresh had higher levels of general happiness, recent satisfaction, and recent enjoyment, respectively. Correspondingly, the OR of 0.36 (95%, CI = 0.15–0.88) and OR of 0.39 (95%, CI = 0.16–0.97) suggest that the participants who reported that the air had strong odor (smelly) had lower levels of satisfaction with how life turned out and satisfaction with standards of living, respectively. Recent happiness is the only measure of +HWB that did not show significant association with perceived odor.

#### 3.4.5. Well-Being and Odor Acceptability

Satisfaction with how life turned out and satisfaction with standards of living were associated with odor acceptability. The OR of 2.16 (95%, CI = 1.18–3.97) and OR of 2.41 (95%, CI = 1.29–4.49) suggest that the participants who reported that odor was highly acceptable had higher levels of satisfaction with how life turned out and satisfaction with standards of living, respectively.

From the two previous sections, we can see that perceived odor was associated with five measures of SWB that represent EWB and +HWB. On the other hand, odor acceptability was associated with only two of measures that represent EWB. This association indicates that residents who lived in areas exposed to strong industrial odors (air has strong odor and low odor acceptability) had lower levels of EWB and +HWB. This lends support to previous findings in the literature, as summarized below.

The number of studies that linked well-being to odor exposure is limited. Some of these studies used psychological stress as a measure of well-being. In a study on health effects of odors from a petroleum refinery in Oakville, Ontario, Luginaah et al. [26] found that the decrease in odor exposure led to decrease in negative perception and concerns. The same study found an association between psychological reaction to general environmental stress and odor exposure. Another study found that hormones released in human body in response to stress were correlated with odorant exposure from a mushroom fertilizer production plant [40]. The association between stress and odor exposure was also confirmed in a cross-sectional study in animal research [41].

Other studies focused on the impact of odor exposure (mainly from animal production) on mental state indices such as depression, anger, confusion, tension, fatigue, and ability to focus. In a study about health and quality of life of residents from North Carolina near intensive livestock operations [77], it was found that residents who live near the swine operations experience reduced quality of life. Schiffman et al. [44] reported an association between unpleasant odors (from large hog operations) and mood state. The residents who were exposed the strong swine odors were most likely to have higher levels of depression, anger, fatigue, confusion, and tension when compared with residents who were not exposed to such odors. This is supported by the findings of Nordin et al. [45], who found that unpleasant odors had a negative impact on the ability to focus. 

Our findings of the association between odor exposure, represented by perceived odor, with EWB, and +HWB agree with the reduction in quality of life and increase in general environmental stress caused by odor exposure found in previous studies [26,40,77]. The association between stress-related indices and well-being is also documented in the literature [78,79]. However, our findings are not in complete agreement with the studies that used mental state indices to measure well-being. These studies showed that odor exposure impacted negative hedonic well-being aspects such as stress, depression, anger, fatigue, confusion, and tension [44,45]. Although our findings showed a decrease in −HWB with the decrease in odor exposure, this association was not statistically significant. In odor exposure studies, the importance of evaluating the SWB based on the three aspects of EWB, +HWB, and −HWB should not be overlooked. Using the three aspects produced different levels of association based on the odor exposure assessment method. While the odor exposure assessed by odor acceptability seem to be associated with evaluative well-being only, the odor exposure assessed by perceived odor was associated with both evaluative and hedonic well-being. 

When interpreting our findings, it could be implied that four of our measures can be chosen to represent SWB (subjective well-being representatives, WB-Rep). Satisfaction with how life turned out, satisfaction with standards of living, recent satisfaction, and recent enjoyment are associated with both employment and perceived odor. They are associated with employment, which is known to be correlated with well-being measures. They are also associated with perceived odor, which is indicative of air quality. On the other hand, negative hedonic measures of SWB (depression, powerlessness, and sadness) may not be good representatives of SWB in communities affected by industrial odors. While they are associated with both employment and age, they did not show a significant association with perceived odor or odor acceptability.

## 4. Additional Analyses

### 4.1. Potential Confounding Variables

Our hypothesis is that people who live in industrial areas experience lower levels of SWB. One might argue that areas surrounding the industrial facilities are often communities of color and/or lower socioeconomic communities. In the context of this argument, residents of those communities have lower levels of well-being because of other factors, such as level of education or income. In other words, the hypothesized association might be influenced by some third confounding variable. In this section, we investigated the effect of potential confounding variables. Diener et al. [38] pointed out that sociodemographic factors and social relationships must be considered when we examine potential confounders in SWB-related studies. Our list of the *target* variables that could be potential confounders includes three binary variables and four ordinal variables. Table 5 details the outcomes of each target variable.

A confounder variable is a variable that was not accounted for (not included in the model), which can bias the model results and lead to incorrect conclusions. In order for a variable to be a confounder, (1) it must be predictive of the dependent variable; (2) it must be correlated with the independent variable; (3) it must be unequally distributed between treatment groups; and (4) it must not be a link in the causal chain [80,81].

The ordinal logistic regression model showed that perceived odor was associated with happiness, satisfaction with how life turned out, satisfaction with standards of living, recent satisfaction, and recent enjoyment. It also showed that odor acceptability was associated with satisfaction with how life turned out and satisfaction with standards of living. The goal was to test whether the target variable correlated with the dependent variables (condition 1) and with the independent variables (condition 2).

Table 6 reports *p*-values from the chi-square test for independence between target variables and variables of interest (both independent and dependent). Except for gender, all target variables are not confounders because they do not meet condition (1). Gender seems to be associated only with perceived odor (*p* = 0.04). When we further test condition (2) for gender, we can see that gender is also associated with happiness (*p* = 0.012), satisfaction with how life turned out (*p* = 0.017), and recent enjoyment (*p* = 0.003). This indicates that gender is a potential confounder of perceived odor, because it is not equally distributed and obviously it is not on the causal chain between perceived odor and the SWB measures. Many studies found that gender is a potential confounder [82,83].

One way to control for confounding effects in analysis is by using a multivariable model (proportional odds regression, in our case), which assesses the hypothesized relationship, and at the same time, adjusting for the potential confounder, age. Consequently, ORs from our model were adjusted for age. The model also has two odor-related variables of interest and two binary demographic variables in order to compare with the literature. Regardless of which of the independent variables is a confounder or an exposure (variable of interest), the model treats all variables the same way [84].

### 4.2. Composite Scoring 

**Figure 4:**
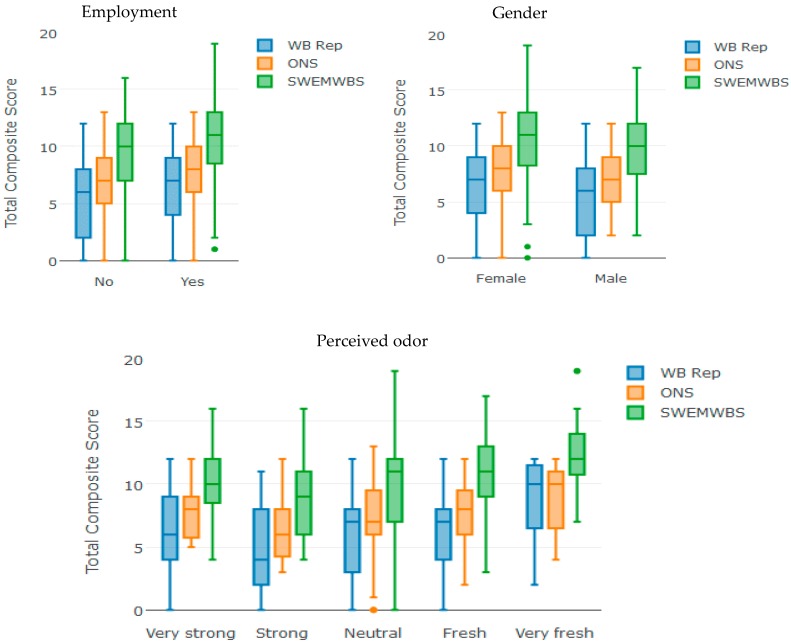
Comparison between subjective well-being representative (WB-Rep), Office for National Statistics (ONS), and Short Warwick–Edinburgh Mental Well-being Scale (SWEMWBS).

We combined our measures of SWB to formulate a single measure and compared it with well-known, reliable, and tested SWB measures already in the literature. From the proportional odds regression conducted in this study, we hypothesized that SWB can be well represented by four measures (satisfaction with how life turned out, satisfaction with standards of living, recent satisfaction, and recent enjoyment). We used the total score (composite score) of the four measures as a subjective well-being representative (WB-Rep). The possible answers of each of the four questions are scored on a scale ranging from 0 to 4. The total composite scores of our WB-Rep, therefore, will be 0 to 16 points [74]. Figure 4 shows a comparison between composite scores of ONS, SWEMWBS, and WB-Rep. The three measures are consistent and show higher levels of well-being for employed and female participants. The three measures also indicate that the well-being levels increase when perceived odor decreases. The WB-Rep positively correlates with ONS (*r* = 0.78, *n* = 326, *p* < 2.2 × 10^−16^), as well as with SWEMWBS (*r* = 0.74, *n* = 326, *p* < 2.2 × 10^−16^).

**Figure 5:**
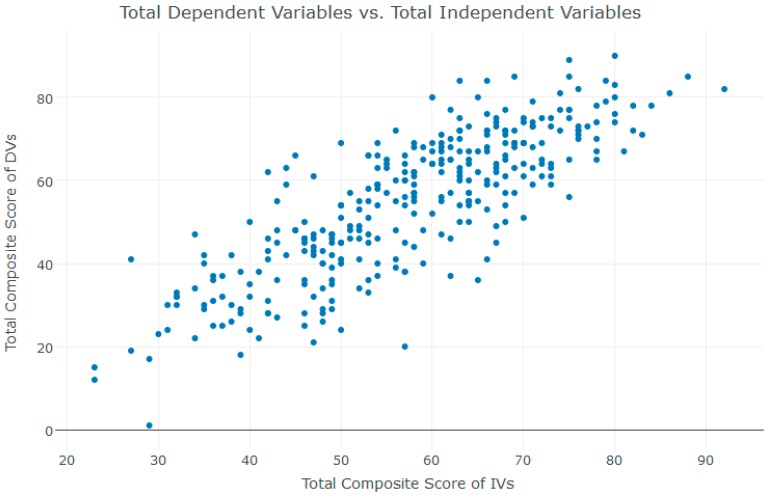
Scatter plot between the total composite scores of SWB explanatory questions and SWB measures. DVs—dependent variables; IVs—independent variables.

A scatter plot between the total composite scores of SWB explanatory questions and SWB measures (*r* = 0.81, *n* = 326, *p* < 2.2 × 10^−16^) is shown in Figure 5. It is worth mentioning that the classification above included only 49 questions (out of 60 questions). The following questions were excluded: the seven demographic questions, a question about odor type, a binary question about employment, a binary question about having someone to discuss matters with, and a question about how many times a participant meets with family and friends. A few other assumptions have been made only for this section’s analysis. To avoid deleting a large number of the dataset, all missing values and values that identified as *Don’t know* were replaced with the value “*2*” [85,86]. The purpose of this demonstration was to show that the classification of NEFs survey questions to explanatory and response questions (measures) can be useful for future studies. 

### 4.3. Seasonal Effect on Well-Being

We analyzed the longitudinal data that were collected by asking participants to take the survey four times during one year (once every three months) in order to see if WB changed from season to season. A total of 55 people took the survey at least twice during the period of time from March 2016 to June 2017. In detail, thirty-six people (65%) took the survey twice, 14 people (26%) took the survey three times, and only 5 people (9%) took the survey four times. The following analysis was conducted on 134 responses. 

We divided the year to four quarters. The first quarter includes January, February, and March. The second includes April, May, and June. The third includes July, August, and September. The fourth includes October, November, and December. There were 38 responses in the first quarter, 41 in the second, 37 in the third, and 18 in the last quarter.

**Figure 6:**
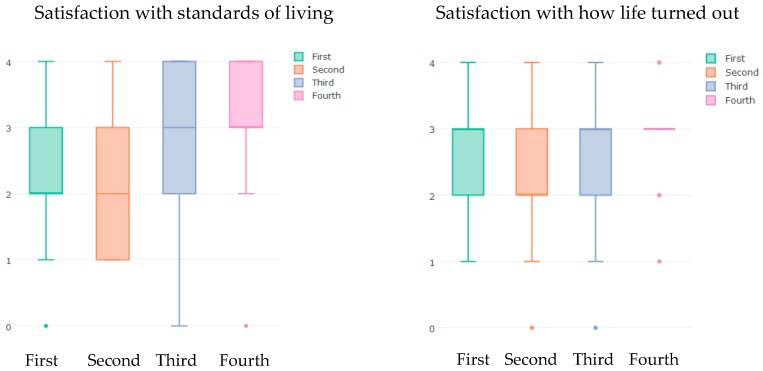
Seasonal variation in satisfaction with standards of living and satisfaction with how life turned out in five communities in Colorado. For both measures, a score of 4 indicates the highest level of satisfaction and a score of 0 indicates the lowest level of satisfaction. *X*-axis in both blots represents seasonal quarters of the year.

Because the sample size in this longitudinal analysis was very small, the contingency table analysis assumption was violated. That is, the number of cell values (expected frequencies) that are less than five (<5) exceeded the conditional limit of 20% (see Table S4). Therefore, all SWB measures were converted to binary variables (e.g., satisfied or not-satisfied). Table 7 shows results from the chi-squared test of independence between season and the binary SWB measures. It can be seen that only two of SWB measures showed a weak association with seasonality—namely, satisfaction with how life turned out ($\chi^{2}\left(3\right)=9.3,\;p<0.03$)—and satisfaction with standards of living ($\chi^{2}\left(3\right)=7.7,\;p<0.05$). Regardless of the weak association and small sample size, these results are not surprising. If SWB had a seasonal pattern, it would be expected that those patterns are more represented by EWB (global) measures than HWB (mood) measures. The chi-squared test does not quantify this weak association. However, boxplots (Figure 6) show that the participants have reported higher levels of the two types of satisfaction during the fourth quarter of the year (October, November, and December). The association between recent depression and seasonality, shown in Table 7, may not be correct because the contingency table analysis assumption was violated (>20%).

### 4.4. Well-Being in the Five Communities

It was hypothesized that the North Denver and Greeley well-being would be different (lower) than the other communities because of the impact of industrial odors. A one-way Analysis of Variance (ANOVA) was conducted to compare the industrial odor levels in the five communities. There was a statistically significant difference between the five communities in regard to perceived odor (F(4,316) = 7.02, P = 2 × 10^−6^). However, there was not a statistically significant difference between communities in odor acceptability levels. North Denver and Greeley clearly stand out as the most affected communities by industrial odors represented by perceived odor levels, as shown by the Tukey Post Hoc Test results in Table 8.

Knowing the significant difference in industrial odor exposure (based on perceived odor levels), we need to answer the following question: are the overall well-being levels in North Denver and Greeley lower than in the other communities? One of the objectives of this work is to compare the well-being levels in the five communities (North Denver, Fort Collins, Fort Lupton, Greeley, and Pueblo).

An ANOVA was used to investigate the differences in the levels of the nine well-being measures between the five communities. The differences were statistically significant in four well-being measures: satisfaction with standards of living (F(4,316) = 2.57, P = 0.04), satisfaction nowadays (F(4,318) = 4.92, P = 0.0007), powerlessness (F(4,307) = 3.104, P = 0.016), and sadness (F(4,310) = 3.798, P = 0.005). Since the ANOVA does not show which specific location (community) is different from the others, a Post Hoc Test, such as the Tukey Post Hoc Test, needs to be used. The results from the Tukey Post Hoc Test are presented in Table 9. All four well-being measures resulting from the ANOVA were lower in Pueblo than in Greeley. Additionally, Pueblo showed lower levels than North Denver in three well-being measures; *satisfaction nowadays, recent powerlessness, and recent sadness.* Lastly, Pueblo’s satisfaction nowadays was also lower than Fort Lupton’s. The difference in well-being levels between North Denver and the other communities was not statistically significant.

The proportional odds regression model showed a strong association between, for example, *satisfaction with standard of living and satisfaction nowadays with perceived odor*. However, from the ANOVA and Tukey Test analysis, the high levels of industrial odors in Greeley and North Denver did not lead to lower levels in the overall well-being in the two communities. In fact, the Tukey test implied that the overall well-being in North Denver is not different than the other communities (except Pueblo). One possibility is that some of the North Denver participants in this study were from neighborhoods that were more likely to be affected by industrial odors, such as Globeville and Elyria Swansea, while other participants were from neighborhoods that were less likely to be affected by industrial odors, such as Sloan Lake and Wheat Ridge. The analysis indicated that Pueblo’s overall well-being levels were lower than all other communities. This might require an urgent, more detailed study to investigate the reasons behind this low level of well-being.

## 5. Conclusions

Odor pollution was identified as a top priority of the community of North Denver. Previous studies that investigated the impact of air pollution in North Denver focused on air pollution sources and adverse health effects, rather than impact on mental well-being. In this study, we investigated whether odors from industrial sources impair the SWB of residents in North Denver and four communities in Colorado. To evaluate SWB in the five communities, nine measures from an online survey were used. The nine measures were grouped into three aspects: evaluative well-being, positive hedonic well-being, and negative hedonic well-being. This classification was based on recent studies and is in good agreement with the results from the PCA analysis. To the best of our knowledge, this approach of evaluating SWB has not been used in previous odor exposure studies. For every SWB measure, an ordinal logistic regression (in particular, a proportional odds regression) was used to quantify the relationship between that particular SWB measure and five independent variables. The five independent variables were employment, age, gender, perceived odor, and odor acceptability. The ordinal logistic regression we used showed that participants who were employed and female participants had higher levels of SWB. Almost all previous studies showed that unemployment had a strong negative impact on SWB. Our finding about the employment and SWB relationship was in good agreement with the literature. The impact of gender on SWB in the literature lacks consistency. However, our findings showed that female participants had higher levels of SWB. We also found that participants who were aged 45 or below had higher levels of recent depression, recent powerlessness, and recent sadness. Regarding the relationship between odor exposure and SWB, which is the aim of this study, we found that the participants who reported that the air is very fresh or odor is highly acceptable had higher levels of SWB. This association indicates that residents who live in areas exposed to strong industrial odors had lower levels of SWB. This lends support to previous findings in the literature, which indicated that unpleasant odors induced annoyance and general psychological stress, and reduced quality of life. While some studies showed that odor exposure (mostly from animal production) had an impact on mood state and ability to focus, our study did not show a significant association between odor exposure and negative hedonic well-being. Using the three aspects of EWB, +HWB, and −HWB in odor exposure studies is important and should not be overlooked. Depending on the odor exposure assessment method, the association between odor exposure could be with one or two SWB aspects. While the odor exposure assessed by odor acceptability seems to be associated with evaluative well-being, the odor exposure assessed by perceived odor was associated with both evaluative and hedonic well-being.

We also found that four of our nine measures can be used to represent SWB in future studies. Two of those measures were evaluative SWB (satisfaction with how life turned out and satisfaction with standards of living) and the other two were positive hedonic SWB measures (satisfaction with life recently and recent enjoyment). The composite score of these four measures (named WB-Rep by us) showed good agreement with reliable, well-known, and tested SWB measures, namely ONS and SWEMWBS. 

Longitudinal analysis showed that evaluative satisfaction was slightly associated with seasonality. Both satisfaction with how life turned out and satisfaction with standards of living slightly increased during the fourth quarter of the year (October to December).

A comparison between the five communities showed that well-being levels in North Denver and Greeley were not significantly different than in Fort Collins or Fort Lupton. The comparison, however, showed that Pueblo had the lowest levels of well-being among all communities.

## Figures and Tables

**Table 1:** Demographic information of the five communities.

Communities	Population	Median Household Income (k)	Average Household Size	Hispanic (%)	Bachelor or Higher (%)
Denver * Globeville Elyria Swansea	600,158 3360 6940	$50.3 k $26.5 k $33.8 k	2.22	31.8 68.7 81.8	42.9 11.2 11.3
Greeley City	92,889	$46.3 k	2.63	36.0	25.8
Fort Lupton City	7377	$50.2 k	3.09	55.0	8.9
Pueblo City	106,595	$34.7 k	2.37	49.8	19.7
Fort Collins City	143,986	$53.8 k	2.37	10.1	51.9

* Denver data presented here are for the entire county of Denver, but the study focuses on the northern neighborhoods of Denver.

**Table 2:** Pattern matrix of four-component principal component analysis (PCA) solution with oblique rotation of 24 well-being measures.

Items of Well-Being	Component			
	1	2	3	4
Satisfaction with how life turned out	0.725			
General happiness	0.721			0.366
Optimistic	0.675			
Satisfaction with life nowadays	0.661			0.381
My life is close to how I would like it to be	0.624			
Satisfaction with standards of living	0.620			0.362
Enjoyed life recently	0.619			
Feeling positive	0.611			
My life valuable	0.584			
Feeling a sense of accomplishment	0.564			−0.308
Recently happy	0.525			
Feeling close to people in my area	0.487		0.307	
Recently sad		0.762		
Recently bored		0.732		
Recently lonely		0.723		
Recently depressed		0.680		
Recently feeling powerless		0.636		
It takes me a long time to get back to normal		0.546		
I am a failure	0.335	0.401		
Recently my sleep has been restless			0.726	
Recently feeling tired			0.720	
Recently I woke up rested	0.355		0.684	
Most people cannot be trusted				0.662
Recently everything I did was an effort	−0.301	0.456	0.339	0.469

Extraction Method: Principal Component Analysis. Rotation Method: Oblimin with Kaiser Normalization.

**Table 3:** Pattern matrix of three-component PCA solution with oblique rotation of the nine selected well-being measures.

The Nine Selected Items of Well-Being	Component		
	1	2	3
General happiness	0.879		
Satisfaction with standards of living	0.867		
Satisfaction with life nowadays	0.857		
Satisfaction with how life turned out	0.830		
Recently feeling powerless		0.904	
Recently sad		0.786	
Recently depressed		0.712	
Recently happy			−0.920
Enjoyed life recently			−0.771

Extraction Method: Principal Component Analysis. Rotation Method: Oblimin with Kaiser Normalization.

**Table 4:** Chi-squared test for independence between all subjective well-being (SWB) measures and the independent variables.

Investigated Variables	Chi-Squared ($\chi^{2})$	Degrees of Freedom	*p* Value ^1^	Contingency Assumption ^2^
Happiness in general				
Employment	11.6	4	0.02 *	<20%
Gender	12.9	4	0.01 *	<20%
Age	4.18	4	0.38	20%
Perceived odor	30.9	16	0.01 *	>20%
Odor acceptability	16.6	8	0.03 *	20%
Satisfaction with how life turned out				
Employment	16.52	4	0.002 *	<20%
Gender	12.1	4	0.02 *	<20%
Age	3.95	4	0.41	20%
Perceived odor	30.4	16	0.02 *	>20%
Odor acceptability	16.9	8	0.03 *	20%
Satisfaction with standards of living				
Employment	11.67	4	0.02 *	<20%
Gender	5.3	4	0.26	<20%
Age	2.44	4	0.66	<20%
Perceived odor	26.8	16	0.04 *	>20%
Odor acceptability	23.0	8	0.003 *	<20%
Satisfaction with life nowadays				
Employment	19.1	4	0.0007 *	<20%
Gender	5.8	4	0.22	<20%
Age	3.84	4	0.43	<20%
Perceived odor	43.6	16	0.0002 *	>20%
Odor acceptability	17.6	8	0.02 *	20%
Recent enjoyment				
Employment	1.69	3	0.6	<20%
Gender	13.7	3	0.003 *	<20%
Age	4.24	3	0.24	<20%
Perceived odor	14.9	12	0.25	>20%
Odor acceptability	3.23	6	0.78	<20%
Recent happiness				
Employment	2.79	3	0.4	<20%
Gender	9.4	3	0.024 *	<20%
Age	3.70	3	0.30	<20%
Perceived odor	12.9	12	0.38	>20%
Odor acceptability	5.22	6	0.52	>20%
Recent depression				
Employment	7.9	3	0.048 *	<20%
Gender	13.6	3	0.004 *	<20%
Age	5.52	3	0.14	<20%
Perceived odor	14.3	12	0.28	20%
Odor acceptability	11.9	6	0.06	<20%
Recent powerlessness				
Employment	13.5	3	0.004 *	<20%
Gender	8.7	3	0.034 *	<20%
Age	8.19	3	0.04 *	<20%
Perceived odor	7.5	12	0.82	>20%
Odor acceptability	1.04	6	0.98	<20%
Recent sadness				
Employment	3.34	3	0.34	<20%
Gender	6.8	3	0.08 *	<20%
Age	5.9	3	0.12	<20%
Perceived odor	8.5	12	0.75	>20%
Odor acceptability	10.5	6	0.1	<20%

^1,^* indicates significance; ^2^ the percentage of number of cells that have expected frequencies less than five to the total number of cells in the contingency table.

**Table 5:** Target variables and their outcomes.

Variable	Type	Outcomes
Employment	Binary	Yes/No
Age	Binary	45 or below/Above 45
Gender	Binary	Male/Female
Education	Ordinal	Below high school/High school graduate/Some college credit, No degree/Bachelor’s degree/Graduate level
Income	Ordinal	Less than 25,000/25,000 to 34,999/35,000 to 49,999/50,000 to 74,999/75,000 to 99,999/100,000 to 149,999/150,000 or more
Marital status	Ordinal	Single, never married/Married or Domestic partnership/Separated/Widowed/Divorced
Race	Ordinal	White/Hispanic or Latino/Other

**Table 6:** Association between target variables and both dependent and independent variables.

Target Variable	Association with Independent Variables		Association with the Dependent Variables				
	Association with Perceived Odor	Association with Odor Acceptability	General Happiness	Satisfaction with How Life Turned Out	Satisfaction with Standards of Living	Satisfaction with Life Nowadays	Enjoy Life Recently
Age	0.20	0.62	0.38	0.41	0.66	0.43	0.24
Gender	0.04	0.095	0.012	0.017	0.26	0.22	0.003
Employment	0.47	0.67	0.02	0.002	0.02	0.0008	0.64
Education	0.74	0.53	0.023	0.008	0.036	0.014	0.86
Income	0.17	0.44	0.061	0.35	0.003	0.18	0.67
Marital status	0.28	0.25	0.014	0.005	0.35	0.0013	0.51
Race	0.31	0.41	0.15	0.62	0.81	0.32	0.67

**Table 7:** Relationship between season and the nine measures of subjective well-being. The results are from the chi-squared test, including *p* value, chi test value, degree of freedom (DOF), and contingency assumption. All well-being (WB) measures are binary variables.

Chi Squared Test for Independence	Happiness in General	Satisfaction with How Life Turned Out	Satisfaction with Standards of Living	Satisfaction with Life Nowadays	Recent Enjoyment	Recent Happiness	Recent Depression	Recent Power-Lessness	Recent Sadness
*p*-value	0.25	0.03	0.05	0.33	0.2	0.07	0.00095	0.38	0.3
$\chi^{2}$	4.1	9.3	7.7	3.4	4.65	7.01	16.4	3.1	3.6
DOF	3	3	3	3	3	3	3	3	3
Contingency assumption	12.5%	12.5%	12.5%	12.5%	12.5%	0%	>20%	0%	12.5%

**Table 8:** The summary table of the Tukey Post Hoc Test results among communities’ perceived odor. Only significant results are presented. ANOVA—Analysis of Variance.

Industrial Odors Measure that Showed Significant Difference between Communities Based on ANOVA	The Two Specific Locations that Showed Significant Different Levels of Industrial Odors Based on Tukey Post Hoc Test		Difference	*p*-Value
	**Location 1**	**Location 2**		
Perceived odor	Greeley	Fort Collins	−0.7 *	0.019
	North Denver	Fort Collins	−0.66	0.045
	Pueblo	Greeley	0.60	0.0002
	Pueblo	North Denver	0.55	0.003

* Negative sign in the difference column indicates that the first community (e.g., Greeley in the first row) has lower levels of perceived odor than the second community (e.g., Fort Collins).

**Table 9:** The summary table of the Tukey Post Hoc Test results among communities’ well-being. Only significant results are presented.

Well-Being Measure that Showed Significant Difference between Communities Based on ANOVA	The Two Specific Locations that Showed Significant Different Levels of well-Being Based on Tukey Post Hoc Test		Difference	*p*-Value
	**Location 1**	**Location 2**		
Satisfaction with standard of living	Pueblo	Greeley	−0.45 *	0.042
Satisfaction Nowadays	Pueblo	Fort Lupton	−0.52	0.04
	Pueblo	Greeley	−0.39	0.03
	Pueblo	North Denver	−0.58	0.0008
Recent Powerlessness	Pueblo	Greeley	−0.33	0.05
	Pueblo	North Denver	−0.38	0.03
Recent Sadness	Pueblo	Greeley	−0.37	0.019
	Pueblo	North Denver	−0.40	0.022

* Negative sign in the difference column indicates that the first community (e.g., Pueblo in the first row) has lower levels of the well-being measure than the second community (e.g., Greeley).

## Supplementary Materials

The following are available online at http://www.mdpi.com/1660-4601/15/6/1091/s1. Survey S1: The Industrial Odors and Well-being Questionaire. Table S1: Office for National Statistics (ONS) Questions. Table S2: The Short Warwick–Edinburgh Mental Well-being Scale (SWEMWBS). Table S3: Social Trust Question. Table S4: Relationship between season and the nine measures of subjective well-being. The results are from the chi-squared test, including p value, chi-test value, degree of freedom, and contingency assumption. WB measure ranges from 0–4 and 1–4.
