# Supplementary material for: The Impact of Industrial Odors on the Subjective Well-Being of Communities in Colorado
Source: Int J Environ Res Public Health. 2018 May 28;15(6):1091. doi: 10.3390/ijerph15061091 (PMC6025584; doi:10.3390/ijerph15061091)
Supplement: Supplementary file 1 [file ijerph-15-01091-s001.pdf]

Supplementary

Survey S1: The Industrial Odors And Wellbeing Questionaire

This Survey consists of two parts:

Part 1: These questions pertain to your well-being

All things considered, how satisfied are you with life as a whole nowadays?

(0 = Extremely dissatisfied) (10 = Extremely satisfied)

0 1 2 3 4 5 6 7 8 9 10 Don’t know

How often do you meet socially with friends, relatives or colleagues?

Never

Less than once a month

Once a month

Several times a month

Once a week

Several times a week

Every day

Don't know

Do you have anyone with whom you can discuss intimate and personal matters?

Yes

No

Don't know

How is your health in general?

Very good

Good

Fair

Bad

Very bad

Don't know

Taking all things together, how happy would you say you are?

0 1 2 3 4 5 6 7 8 9 10 Don’t know

I'm always optimistic about my future.

Agree strongly

Agree

Neither agree nor disagree

Disagree

Disagree strongly

Don't know

At times I feel as if I am a failure.

Agree strongly

Agree

Neither agree nor disagree

Disagree

Disagree strongly

Don't know

In general, I feel very positive about myself.

Agree strongly

Agree

Neither agree nor disagree

Disagree

Disagree strongly

Don't know

On the whole, my life is close to how I would like it to be.

Agree strongly

Agree

Neither agree nor disagree

Disagree

Disagree strongly

Don't know

How much of the time during the past week have you felt depressed?

None or almost none of the time

Some of the time

Most of the time

All or almost all of the time

Don't know

How much of the time during the past week have you felt that everything you did was an effort?

None or almost none of the time

Some of the time

Most of the time

All or almost all of the time

Don't know

How much of the time during the past week has your sleep been restless?

None or almost none of the time

Some of the time

Most of the time

All or almost all of the time

Don't know

How much of the time during the past week were you happy?

None or almost none of the time

Some of the time

Most of the time

All or almost all of the time

Don't know

How much of the time during the past week have you felt lonely?

None or almost none of the time

Some of the time

Most of the time

All or almost all of the time

Don't know

How much of the time during the past week have you enjoyed life?

None or almost none of the time

Some of the time

Most of the time

All or almost all of the time

Don't know

How much of the time during the past week have you felt sad?

None or almost none of the time

Some of the time

Most of the time

All or almost all of the time

Don't know

How much of the time during the past week could you not get going?

None or almost none of the time

Some of the time

Most of the time

All or almost all of the time

Don't know

How much of the time during the past week have you had a lot of energy?

None or almost none of the time

Some of the time

Most of the time

All or almost all of the time

Don't know

How much of the time during the past week have you felt tired?

None or almost none of the time

Some of the time

Most of the time

All or almost all of the time

Don't know

How much of the time during the past week have you been absorbed in what you were doing?

None or almost none of the time

Some of the time

Most of the time

All or almost all of the time

Don't know

How much of the time during the past week have you felt bored?

None or almost none of the time

Some of the time

Most of the time

All or almost all of the time

Don't know

How much of the time during the past week have you felt rested when you woke up in the morning?

None or almost none of the time

Some of the time

Most of the time

All or almost all of the time

Don't know

I feel I am free to decide how to live my life.

Agree strongly

Agree

Neither agree nor disagree

Disagree

Disagree strongly

Don't know

In my daily life, I seldom have time to do the things I really enjoy.

Agree strongly

Agree

Neither agree nor disagree

Disagree

Disagree strongly
Don't know

In my daily life I get very little chance to show how capable I am.
Agree strongly
Agree
Neither agree nor disagree
Disagree
Disagree strongly
Don't know
Most days I feel a sense of accomplishment from what I do.
Agree strongly
Agree
Neither agree nor disagree
Disagree
Disagree strongly
Don't know
When things go wrong in my life, it generally takes me a long time to get back to normal.
Agree strongly
Agree
Neither agree nor disagree
Disagree
Disagree strongly
Don't know

My life involves a lot of physical activity.
Agree strongly
Agree
Neither agree nor disagree
Disagree
Disagree strongly
Don't know

How satisÞed are you with how your life has turned out so far?
(0 = Extremely dissatisÞed) (10 = Extremely satisÞed)
0 1 2 3 4 5 6 7 8 9 10 Don’t know

How satisÞed are you with your present standard of living?
(0 = Extremely dissatisÞed) (10 = Extremely satisÞed)
0 1 2 3 4 5 6 7 8 9 10 Don’t know

Most people can be trusted, or you can't be too careful?

(0 = Can't be too careful) (10 = Most people can be trusted)

0 1 2 3 4 5 6 7 8 9 10 Don't know

How much of the time spent with your immediate family is enjoyable?

(0 = None of the time) (6 = All of the time)

0 1 2 3 4 5 6 Don't know

How much of the time spent with your immediate family is stressful?

(0 = None of the time) (6 = All of the time)

0 1 2 3 4 5 6 Don't know

To what extent do you get a chance to learn new things?

4/10/2016 Odors and Wellbeing

(0 = Not at all) (6 = A great deal)

0 1 2 3 4 5 6 Don't know

To what extent do you feel that people in your local area help one another?

(0 = Not at all) (6 = A great deal)

0 1 2 3 4 5 6 Don't know

To what extent do you feel that people treat you with respect?

(0 = Not at all) (6 = A great deal)

0 1 2 3 4 5 6 Don't know

To what extent do you feel that people treat you unfairly?

(0 = Not at all) (6 = A great deal)

0 1 2 3 4 5 6 Don't know

To what extent do you feel that you get the recognition you deserve for what you do?

0 1 2 3 4 5 6 Don't know

I generally feel that what I do in my life is valuable and worthwhile.

Agree strongly

Agree

Neither agree nor disagree

Disagree

Disagree strongly

Don't know

There are people in my life who really care about me.

Agree strongly

Agree

Neither agree nor disagree

Disagree

Disagree strongly

Don't know

I feel close to the people in my local area.

Agree strongly

Agree

Neither agree nor disagree

Disagree

Disagree strongly

Don't know

Are you currently in paid work of any kind?

Yes

No

All things considered, how satisÞed are you with your present job?

(0 = Extremely dissatisÞed) (10 = Extremely satisÞed)

0 1 2 3 4 5 6 7 8 9 10 Don’t know

How satisÞed are you with the balance between the time you spend on your paid work and the time you spend on other aspects of your life?

(0 = Extremely dissatisÞed) (10 = Extremely satisÞed)

0 1 2 3 4 5 6 7 8 9 10 Don’t know

How much of the time do you Þnd your job interesting?

(0 = None of the time) (6 = All of the time)

0 1 2 3 4 5 6 Don’t know

How much of the time do you Þnd your job stressful?

(0 = None of the time) (6 = All of the time)

0 1 2 3 4 5 6 Don’t know

How likely would you say it is that you will become unemployed in the next 12 months?

Very likely

Likely

Not very likely

Not at all likely

Don't know

Considering all my efforts and achievements in my job, I feel I get paid appropriately?

Agree strongly

Agree

Neither agree nor disagree

Disagree

Disagree strongly

Don't know

Part 2

These questions pertain to air quality near your place

Do you think the air is fresh near your home?

Very fresh

Fresh

Neutral

Strong

Very strong

Is the odor near your home acceptable?

10 = clean outdoor air (mountain) .... 0 = very dirty air (irritating - China's pollution, if familiar)

0 1 2 3 4 5 6 7 8 9 10

How is the odor intensity near your home on a scale from no odor to overwhelming odor?

No odor

Slight

Moderate

Strong

Very strong

Overwhelming

Does the odor affect your outdoor activities, using the backyard or opening windows?

Never

I notice odors but they don't affect our life

Sometimes they affect our life

Often they affect our life

I cannot use my backyard, open windows, or do any outdoor activities because of them.

Don't know

What does the odor smell like?

Choose only one.

Dog food

Natural gas

Car exhaust

Asphalt/Creosote/Tar

Sewer

Animal (rendering/dead)

Livestock

Chemical

rotten eggs

Other

Don't smell odors

Does any of your family members have any of these health symptoms?

(You may choose more than one)

Problems breathing

Problems sleeping

Skin irritations

Dizziness

High blood pressure

Headaches

Nervousness

Runny nose

Eye irritation

Other

No symptoms

How old are you?

18-25

26-35

36-45

46-55

56-65

66-75

76-85

86 or more

Prefer not to disclose

What is your gender?

Female

Male

Prefer not to disclose

Ethnicity origin (or Race): Please specify your ethnicity?

White

Hispanic or Latino

Black or African American

Native American or American Indian

Asian / Pacific Islander

Other

Education: What is the highest degree or level of school you have completed?

No schooling completed

Nursery school to 8th grade

Some high school, no diploma

High school graduate, diploma or equivalent (for example: GED)

Some college credit, no degree

Bachelor's degree or Associate degree

Master's degree

Professional degree

Doctorate degree

Marital Status: What is your marital status?

Single, never married

Married or domestic partnership

Widowed

Divorced

Separated

Number of children: how many children do you have?

0

1

2

3

4

More than 4

Household income: What is your total household income during the past 12 months?

Less than $25,000

$25,000 to $34,999

$35,000 to $49,999

$50,000 to $74,999

$75,000 to $99,999

$100,000 to $149,999

$150,000 or more

Where do you live currently? (optional)

Street name and/or building number (optional)

Please type your email address (or the code you received from us):

We will use this email to send you report requests and contact you to send the $5 gift card

Table Supplementary1: Office for National Statistics (ONS) Questions

Question	Range
Overall, how satisfied are you with your life nowadays?	0 to 10
Overall, how happy did you feel yesterday?	0 to 10
Overall, how anxious did you feel yesterday?	0 to 10
Overall, to what extent do you feel the things you do in your life are worthwhile?	0 to 10

Table Supplementary2: The Short Warwick-Edinburgh Mental Well-being Scale (SWEMWBS)

Question	Range
I've been feeling optimistic about the future	None of the time, Rarely, Some of the time, Often, All of the time
I've been feeling useful	None of the time, Rarely, Some of the time, Often, All of the time
I've been feeling relaxed	None of the time, Rarely, Some of the time, Often, All of the time
I've been dealing with problems well	None of the time, Rarely, Some of the time, Often, All of the time
I've been thinking clearly	None of the time, Rarely, Some of the time, Often, All of the time
I've been feeling close to other people	None of the time, Rarely, Some of the time, Often, All of the time
I've been able to make up my mind about things	None of the time, Rarely, Some of the time, Often, All of the time

Table Supplementary3: Social Trust

Question	Range
Generally speaking, would you say that most people can be trusted, or that you can't be too careful in dealing with people?	Please give a score of 0 to 10, where 0 means you can't be too careful and 10 means that most people can be trusted.

Table Supplementary4: Relationship between season and the nine measures of subjective wellbeing. The results are from Chi-squared test including p value, chi test value, degree of freedom and contingency assumption. WB measure range from 0-4 and 1-4.

	**Happiness in general**	**Satisfaction with how life turned out**	**Satisfaction with standards of living**	**Satisfaction with life nowadays**	**Recent enjoyment**	**Recent happiness**	**Recent depression**	**Recent power-lessness**	**Recent sadness**
**p-value**	0.55	0.19	0.01	0.42	NA	0.1	0.02	0.32	0.13
χ^2	10.7	15.97	25.7	12.39	12	12	23.55	13.68	13.85
**DOF**	12	12	12.	12	NA	18.47	12	12	9
**# cells less < 5**	>20%	>20%	>20%	>20%	>20%	>20%	>20%	>20%	>20%
